# Sulfide-dependent Photoautotrophy in the Filamentous Anoxygenic Phototrophic Bacterium, *Chloroflexus aggregans*

**DOI:** 10.1264/jsme2.ME19008

**Published:** 2019-08-08

**Authors:** Nanako Kanno, Shin Haruta, Satoshi Hanada

**Affiliations:** 1 Department of Biological Sciences, Graduate School of Science, Tokyo Metropolitan University 1–1 Minami-Osawa, Hachioji, Tokyo 192–0397 Japan

**Keywords:** Filamentous anoxygenic phototrophic bacteria, *Chloroflexus aggregans*, photoautotrophy, sulfide oxidation, hot spring

## Abstract

*Chloroflexus aggregans* is a thermophilic filamentous anoxygenic phototrophic bacterium frequently found in microbial mats in natural hot springs. *C. aggregans* often thrives with cyanobacteria that engage in photosynthesis to provide it with an organic substrate; however, it sometimes appears as the dominant phototroph in microbial mats without cyanobacteria. This suggests that *C. aggregans* has the ability to grow photoautotrophically. However, photoautotrophic growth has not been observed in any cultured strains of *C. aggregans*. We herein attempted to isolate a photoautotrophic strain from *C. aggregans*dominated microbial mats in Nakabusa hot spring in Japan. Using an inorganic medium, we succeeded in isolating a new strain that we designated “ACA-12”. A phylogenetic analysis based on 16S rRNA gene and 16S-23S rRNA gene internal transcribed spacer (ITS) region sequences revealed that strain ACA-12 was closely related to known *C. aggregans* strains. Strain ACA-12 showed sulfide consumption along with autotrophic growth under anaerobic light conditions. The deposited elemental sulfur particles observed by microscopy indicated that sulfide oxidation occurred, similar to that in photoautotrophic strains in the related species, *C. aurantiacus*. Moreover, we found that other strains of *C. aggregans*, including the type strain, also exhibited a slight photoautotrophic growing ability, whereas strain ACA-12 showed the fastest growth rate. This is the first demonstration of photoautotrophic growth with sulfide in *C. aggregans*. The present results strongly indicate that *C. aggregans* is associated with inorganic carbon incorporation using sulfide as an electron donor in hot spring microbial mats.

The *Chloroflexus* species are thermophilic filamentous anoxygenic phototrophic bacteria generally found in neutral to alkaline hot springs. In these natural hot springs, *Chloroflexus* organisms form microbial mats with cyanobacteria (2, 15), and appear to grow essentially photoheterotrophically or chemoheterotrophically using organic substrates derived from neighboring cyanobacteria. All isolated strains belonging to the genus *Chloroflexus* show good growth in both of these heterotrophic conditions (7, 13). However, previous studies reported the growth of *Chloroflexus* strains in microbial mats independent of cyanobacteria (1, 10, 17), suggesting that these strains have the ability to support themselves by photoautotrophy.

In Nakabusa hot spring (Nagano Prefecture, Japan), *Chloroflexus*-predominated microbial mats without cyanobacteria are frequently found at approximately 65°C (4, 17). Cultureindependent studies based on 16S rRNA gene sequence analyses revealed that the closest species of this *Chloroflexus* was *Chloroflexus aggregans* (4, 17, 23). The water at Nakabusa hot spring contains sulfide (~0.3 mM) (22), which supports the growth of *C. aggregans*. In an *in vitro* experiment using the Nakabusa *C. aggregans* mat, Kubo *et al.* (17) reported that bicarbonate stimulated sulfide consumption by the microbial mat under anaerobic light conditions. This finding suggested that this *C. aggregans* had the ability to grow photoautotrophically using sulfide as an electron donor.

In the genus *Chloroflexus*, two strains, *i.e*., *C. aurantiacus* strain OK-70-fl (14, 24) and *Chloroflexus* sp. strain MS-G (28), have been reported to show photoautotrophic growth with sulfide. In strain OK-70-fl, photoautotrophic growth was also achieved with hydrogen as an electron donor instead of sulfide (14). The photoautotrophy of *Chloroflexus* sp. strain MS-G was observed in anaerobic agar-deep cultures using medium supplemented with sulfide and bicarbonate as the sole electron and carbon sources, respectively (28).

However, photoautotrophic growth with sulfide has not yet been demonstrated in other strains in the genus *Chloroflexus*, including the type strains of three known species: *C. aurantiacus* J-10-fl^T^, *C. aggregans* MD-66^T^, and *C. islandicus* isl-2^T^ (7, 12). Genomic analyses of these three species have provided evidence of their autotrophic ability with sulfide, including the presence of a gene set that is related to one of the carbon fixation pathways (the 3-hydroxypropionate pathway) and a gene for sulfide quinone oxidoreductase (SQR), which mediates electron transport from sulfide to menaquinone (6, 16, 27, 28).

In the present study, we attempted to isolate a photoautotrophic strain of *C. aggregans* from Nakabusa hot spring, and successfully obtained strain ACA-12. The results of our phylogenetic analysis and the physiological properties of this strain strongly suggest that strain ACA-12 is a new strain of *C. aggregans*. We herein describe the photoautotrophic properties and genomic traits of strain ACA-12 and compare them with those of other *C. aggregans* strains that are known as photoheterotrophic organisms.

## Materials and Methods

### Bacterial strains

*C. aggregans* MD-66^T^ (=DSM 9485^T^) was obtained from the Deutsche Sammlung von Mikroorganismen und Zellkulturen (DSMZ, Braunschweig, Germany). *C. aggregans* strain NBF was previously isolated from Nakabusa hot spring (21). These strains were maintained photoheterotrophically in modified PE medium (pH 7.5) containing (L^−1^) 0.1 g sodium glutamate, 0.1 g disodium succinate, 0.1 g sodium acetate, 0.1 g yeast extract (Nihon Seiyaku, Tokyo), 0.1 g Bacto casamino acids (Difco Laboratories, Detroit, MI, USA), 0.1 g Na_2_S_2_O_3_·5H_2_O, 0.1 g (NH_4_)_2_SO_4_, 75 mg KH_2_PO_4_, 78 mg K_2_HPO_4_, 0.2 mL of a vitamin mixture (11), and 1 mL of a basal salt solution (11). Cultures were kept in screw-capped tubes that were completely filled with the same medium and incubated at 55°C under incandescent illumination (approximately 230 W m^−2^).

### Sampling site and isolation

*Chloroflexus*-dominated microbial mats are always found in Nakabusa hot spring in Nagano Prefecture, Japan (36° 23′ 15N″, 137° 45′ 00E″, elevation of 1,500 m). The thickness of these mats that developed at 65°C is approximately 3 mm. Sampling was performed on November 4, 2016. Each single piece of the collected mat (approximately 10 mg wet weight) was incubated in a 100-mL vial containing a 40-mL enrichment medium anaerobically at 55°C under illumination from an incandescent lamp.

The enrichment medium (pH 7.0) was prepared as follows. The vial containing the solution consisted of (L^−1^) 0.5 g Na_2_S_2_O_3_·5H_2_O, 0.5 g (NH_4_)_2_SO_4_, 0.75 g KH_2_PO_4_, 0.78 g K_2_HPO_4_, 1 mL of a vitamin mixture (11), and 10 mL of a basal salt solution (11). The solution was gassed with a mixture of N_2_ and CO_2_ (80%:20% [v/v]) and then autoclaved. The gas phase of the vials was replaced with the same gas composition mixture. Before the inoculation, a filter-sterilized NaHCO_3_ solution (final concentration, 50 mM) and a Na_2_S solution (final concentration, 0.4 mM) were added to the vial.

Strain ACA-12 was isolated from the enrichment culture using an agar plate. An aliquot of the enrichment culture was streaked on an agar plate of modified PE medium (see above) supplemented with 1.5% Bacto agar (Difco Laboratories), and the inoculated plate was incubated under aerobic dark conditions at 55°C. Under these conditions, *C. aggregans* grows by oxygen respiration. These conditions also allow contaminated heterotrophic bacteria to grow and make individual colonies, which facilitates the isolation and purity check of *C. aggregans* strains. The following bacterial properties were investigated: cell morphology was examined under phase-contrast microscopy; the presence of bacteriochlorophyll (BChl) *c* and *a* (666 and 770 nm, respectively) by absorption spectral observations of the pigment extract (acetone-methanol, 7:2 [v:v]); and gliding motility on agar plates.

### Growth conditions for the photoautotrophic growth experiment

The cells of *C. aggregans* strains ACA-12, MD66^T^, and NBF were initially pre-cultured photoheterotrophically. Pre-cultures were serially transferred to the inorganic medium twice for the photoautotrophic growth experiment. The inorganic medium (pH 7.0) for the growth experiment was prepared as follows. The vial containing the solution consisting of (L^−1^) 0.1 g (NH_4_)_2_SO_4_, 0.15 g KH_2_PO_4_, 0.16 g K_2_HPO_4_, 0.2 mL of a vitamin mixture (11), and 2 mL of a basal salt solution (11) was gassed with a mixture of N_2_ and CO_2_ (80%:20% [v/v]) and then autoclaved. Prior to the inoculation, the filter-sterilized NaHCO_3_ solution (final concentration, 50 mM) and Na_2_S solution (final concentration, 0–1.5 mM) were added to the vial.

Cells were pre-cultured photoheterotrophically in modified PE medium at 55°C for 48 h under anaerobic conditions. Cells were harvested by centrifugation (13,982×*g*, 4°C, 5 min) and then washed once with the wash solution containing (L^−1^) 0.75 g KH_2_PO_4_, 0.78 g K_2_HPO_4_, 1 mL of a vitamin mixture (11), and 10 mL of a basal salt solution (11) to remove organic compounds. After washing, cells were re-suspended in 500 μL of the inorganic medium. These cell suspensions were inoculated into 30-mL test tubes containing 10 mL of the inorganic medium with 0.5 mM sulfide in order to achieve parallel cell concentrations (OD660≒0.1), and they were then incubated at 55°C under anaerobic light conditions for 48 h. An aliquot (100 or 400 μL) of cells grown in the inorganic medium was transferred into a 30-mL test tube (for the growth estimation) or 100-mL vial (to measure sulfide concentrations) supplemented with 0–1.5 mM sulfide.

To estimate cell concentrations in the culture, we harvested ACA-12 cells in the tubes onto a filter membrane (0.2-μm pore size, hydrophilic polycarbonate membrane, 25 mm diameter) and resuspended them in 1 mL of phosphate-buffered saline (PBS) (pH ~7.4) with 0.1% Tween 80. Cells were dispersed by sonication (15-s sonication and 15-s interval, 20 KHz/160 W) at 4°C for 10 min in a sonication bath (Bioruptor UCD-200T; Cosmobio, Tokyo). BChl *c* concentrations were assessed by extraction with acetone-methanol as follows. One hundred microliters of the cell suspension was added to 900 μL of acetone-methanol (7:2). The mixture was centrifuged (13,982×*g*, 4°C, 5 min) and the supernatant was collected. The absorption spectrum was recorded between 400 and 1,000 nm using a UV-1800 spectrophotometer (Shimadzu, Kyoto, Japan). To calculate BChl *c* (absorbance at 666 nm) concentrations, we used the extinction coefficient of 74 mM^−1^ cm^−1^ (5). Tubes were examined in triplicate for this experiment.

We used the *in vivo* absorption of BChl *c* to estimate the growth curves of strain MD-66^T^ and NBF. The *in vivo* spectra of the bacterial cultures were recorded by a UV-1800 spectrophotometer (Shimadzu). To cancel the effects of turbidity from extracellular elemental sulfur, we used the value of the quadratic differential of the absorption spectra at 740 nm in the calculation. The smoothing of the original spectrum and the quadratic differential of the absorption spectrum were calculated by the Convolution (Savitzky-Golay) method using UVProve 2.42 software (Shimadzu).

### Sulfide concentrations in the culture

To assess sulfide concentrations in the culture, we prepared a vial separately from those used in the measurement of the growth curve. The sulfide concentration in each culture was measured as follows: 400 μL of the culture was periodically collected from the vials during cultivation and immediately mixed with 400 μL of 0.1 M sodium carbonate—sodium bicarbonate buffer solution (pH 10.0) to prevent hydrogen sulfide from evaporating, and cells were removed by centrifugation at 13,982×*g* for 5 min. The supernatant (150 μL) was collected and fixed with 150 μL of 1.8 mM zinc acetate, and stored at 4°C until analyzed. Sulfide concentrations were assessed by the methylene blue formation method according to Cline (3). The experiment was performed in triplicate.

### Analysis of the 16S rRNA gene, the ITS region sequence, and the type II sulfide quinone oxidoreductase (SQR) gene sequence

We used a Wizard Genomic DNA Purification kit (Promega, Madison, WI) to extract the genomic DNA of strain ACA-12. The 16S rRNA gene of strain ACA-12 was amplified using the 27F (5′-AGAGTTTGATCMTGGCTCAG-3′) and 1492R (5′-TACGGY TACCTTGTTACGACTT-3′) primers (18) with Ex-*Taq* polymerase (Takara, Kusatsu, Japan). Polymerase chain reaction (PCR) amplification was performed by a T100 Thermal Cycler (Bio-Rad Laboratories, Hercules, CA) under the following conditions: initial denaturation at 94°C for 3 min; 30 cycles of denaturation at 94°C for 30 s, primer annealing at 55°C for 45 s, and extension at 72°C for 1 min; and terminal elongation at 72°C for 4 min.

The 16S-23S rRNA gene internal transcribed spacer (ITS) region sequence of strain ACA-12 was amplified using a forward primer (5′-GACACACACGCTACAATGGC-3′) and reverse primer (5′-TGCCCCATTCGGAAATCTCC-3′). The PCR primer set was designed based on the 16S–23S rRNA gene information of strain MD-66^T^ in the present study. PCR amplification was performed under initial denaturation at 95°C for 3 min; 35 cycles of denaturation at 94°C for 1 min, primer annealing at 55°C for 1 min, and extension at 72°C for 1 min; and terminal elongation at 72°C for 3 min.

The SQR sequence of strain ACA-12 was amplified using a forward primer (5′-AAGCCGATTACATCCCACCG-3′) and reverse primer (5′-AACGGGAAGCTCTCTTTGGG-3′). The PCR primer set targeting the majority of the open reading frame (ORF) was designed according to the gene information of strain NBF (LC439416) in the present study. PCR amplification was performed under initial denaturation at 94°C for 2 min; 30 cycles of denaturation at 94°C for 30 s, primer annealing at 60°C for 30 s, and extension at 72°C for 1 min; and a terminal elongation at 72° C for 5 min.

These PCR products were sequenced by the Big Dye Terminator v3.1 Sequencing kit (Applied Biosystems, Foster City, CA) with an ABI3130xl Genetic Analyzer (Applied Biosystems).

### Nucleotide sequence accession numbers

The partial 16S rRNA gene, ITS region, and SQR gene sequences obtained in the present study have been deposited in the DDBJ/EMBL/GenBank nucleotide sequence databases with the following accession numbers: *C. aggregans* ACA-12, LC438939, LC439412, and LC439413; *C. aggregans* NBF, LC439414, LC439415, and LC439416.

### Genomic DNA fingerprinting

The rep-PCR oligonucleotide primers evaluated in the present study were ERIC 1R (5′-ATGTAAGCTCCTGGGGATTCAC-3′) and ERIC 2 (5′-AAGTAAGTGACTGGGGTGAGCG-3′), BOXA1R (5′-CTACGGCAAGGCGACGCTGACG-3′), and (GTG)5 (5′-GTG GTGGTGGTGGTG-3′) (9, 29). The 25-μL PCR mixture contained 50 ng of genomic DNA, 1X Ex *Taq* Buffer (Takara, Otsu, Japan), dNTP mixture (0.2 mM each), 0.4 μM of each primer, and 1.25 U of Takara Ex *Taq* Hot Start Version (Takara). PCR amplification was performed under initial denaturation at 94°C for 3 min; 4 cycles of denaturation at 95°C for 5 min, primer annealing at 40°C for 5 min, and extension at 72°C for 5 min; 30 cycles of denaturation at 94°C for 1 min, primer annealing at 55°C for 1 min, and extension at 72°C for 2 min; and terminal elongation at 72°C for 10 min. Five microliters of the PCR reaction was analyzed on a 1.5% agarose gel. DNA bands were stained with GelGreen (Biotium, Hayward, CA) and visualized on an LED transilluminator (Wako, Osaka, Japan).

## Results

### Genetic characterization of the newly isolated strain, ACA-12

Strain ACA-12 was isolated from Nakabusa hot spring under photoautotrophic conditions using sulfide as the sole electron source. The isolate showed similar phenotypic properties to those of *C. aggregans* strain MD-66^T^, such as a filamentous morphology (Fig. S1) and cell aggregation ability in liquid media (Fig. S2). The absorption spectrum of the extracted pigments indicated that strain ACA-12 contained BChl *c* and *a* (data not shown), similar to other *Chloroflexus* strains (7, 11, 24).

Phylogenetic analyses based on the 16S rRNA gene and ITS region sequences revealed that the isolate ACA-12 was closely related to *C. aggregans* (Table 1). Strain ACA-12 showed 98.9% identity with *C. aggregans* MD-66^T^ in the 16S rRNA gene sequences and 100% identity with *C. aggregans* strain NBF in the sequences of both the 16S rRNA gene and ITS region (Table 1).

**Fig. 1:**
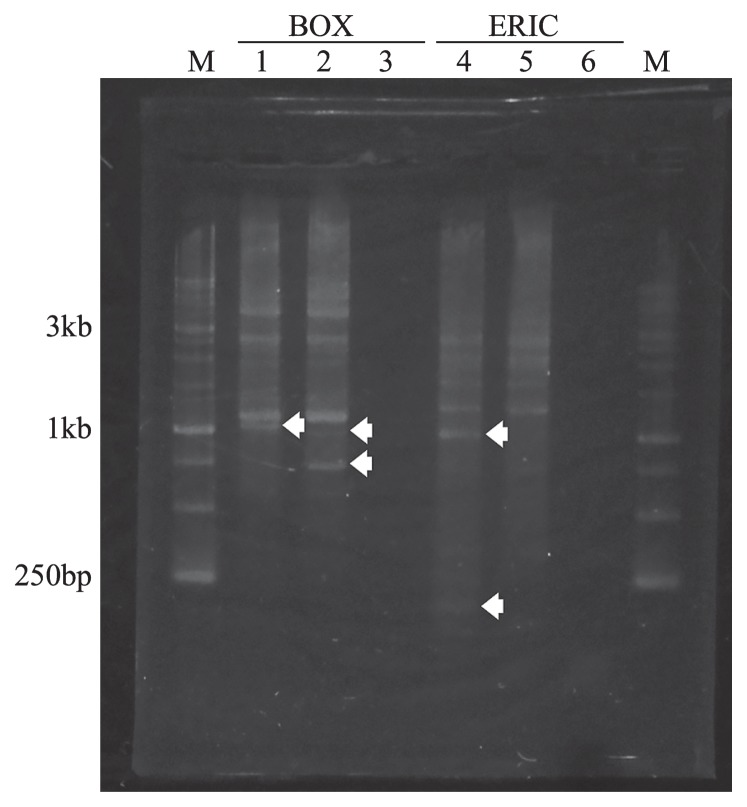
Genomic fingerprinting of *C. aggregans* ACA-12 and NBF by BOX-PCR and ERIC-PCR. Lane 1, BOX-PCR pattern of strain ACA-12; lane 2, BOX-PCR pattern of strain NBF; lane 3, negative control (containing all components, except for DNA) of BOX-PCR; lane 4, ERIC-PCR pattern of strain ACA-12; lane 5, ERIC-PCR pattern of strain NBF; lane 6, negative control of ERIC-PCR; M, ExcelBand 1KB (0.25–10 kb) DNA Ladder (DM3100, SMOBIO TECHNOLOGY, Tokyo, Japan). White arrows show specific bands for each strain.

Strain ACA-12 and *C. aggregans* strain NBF were isolated from the same hot spring in Japan, Nakabusa hot spring, and share phenotypic characteristics, such as their morphology and pigment compositions. Although we were unable to differentiate between these two strains in the analysis based on their 16S rRNA genes and ITS sequences, repetitive-sequence-based PCR DNA fingerprinting (Rep-PCR) clearly distinguished the isolate from strain NBF (8, 25, 26, 30). The PCR amplification of extracted genomic DNAs with BOX or ERIC primers revealed that ACA-12 and NBF each had strain-specific patterns of amplification products (Fig. 1).

BOX-PCR amplification with degenerate primers generated fingerprint patterns with six or seven identifiable bands per strain (Fig. 1). These bands ranged in size between 0.2 and 6.0 kb. Strain ACA-12 and strain NBF shared prominent bands (at approximately 6.0, 4, 2.5, 2, and 1.2 kb). Strain ACA-12 had a specific band (arrow in lane 1, Fig. 1) just below the common band of 1.2 kb. Strain NBF has two specific bands at approximately 1.0 kb and 750 b. Fingerprints obtained by ERIC-PCR also showed the strain-specific patterns of amplification products (Fig. 1). The fingerprint pattern of each strain had seven or nine distinguishable bands, and the bands ranged in size between 0.2 and 4.0 kb. Strains ACA-12 and NBF shared prominent bands (at approximately 4.0 kb, 2.5 kb, 2 kb, 1.5 kb, 1.3 kb, 750 b, and 400 b). Strain ACA-12 had two additional bands at approximately 1.0 kb and below 250 b, whereas these bands were not found in strain NBF. The same band patterns in BOX-PCR and ERIC-PCR were obtained in a repeated experiment.

The genomes of strains MD-66^T^ and NBF contained the gene encoding a Type II sulfide:quinone oxidoreductase (SQR). SQR is a membrane-associated protein that catalyzes the oxidation of sulfide to elemental sulfur and transfers electrons to the membrane quinone pool (20). We amplified a part of the sequence of SQR from the genome of ACA-12 using a PCR primer that was designed according to the gene information of strain NBF. Although the 16S rRNA gene sequences of strains ACA-12 and NBF were different from that of strain MD-66^T^ (1.1% difference), the SQR amino acid sequences of these three strains showed 100% identity (Table 1).

### Photoautotrophic growth with sulfide in *C. aggregans* strain ACA-12

**Fig. 2:**
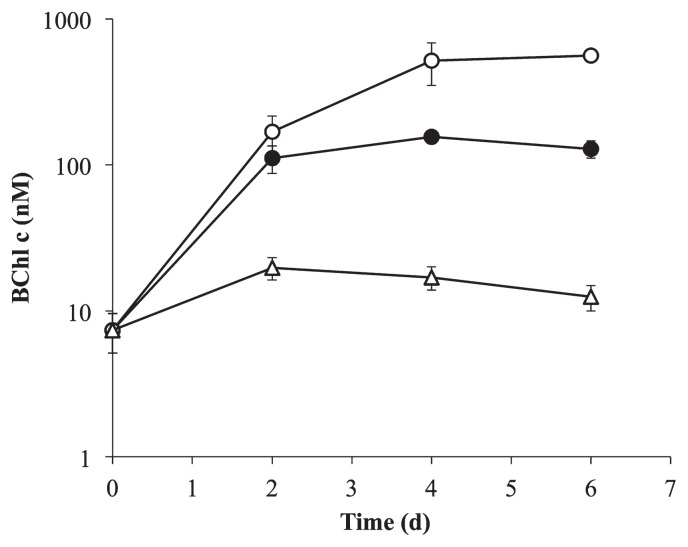
Influence of sulfide concentrations on the photoautotrophic growth of *C. aggregans* strain ACA-12. Closed circle, the culture containing 0.5 mM sulfide; open circle, the culture containing 1.5 mM sulfide; open triangle, the culture without an electron donor. In each case, all cells in the tube were harvested and the pigment was extracted. Data are mean values of triplicate trials±standard deviation.

The photoautotrophic growth of strain ACA-12 was observed in the inorganic medium containing 0.5 or 1.5 mM sulfide (Fig. 2). After a 4-d incubation, the amounts of BChl *c* in media with 0.5 and 1.5 mM sulfide increased by 21- and 70-fold, respectively. Without sulfide, a sufficient increase was not observed. Since a linear correlation was observed between the production of BChl *c* and total cellular protein (see Fig. S3), it is reasonable that the increase in BChl *c* indicated cell reproduction. The growth rate (doublings h^−1^) based on the BChl *c* concentration was calculated from the exponential growth data plotted against time (Fig. S3), and the growth rate was approximately 0.080 doublings h^−1^ when the medium was supplemented with 1.5 mM sulfide (Table 2).

**Fig. 3:**
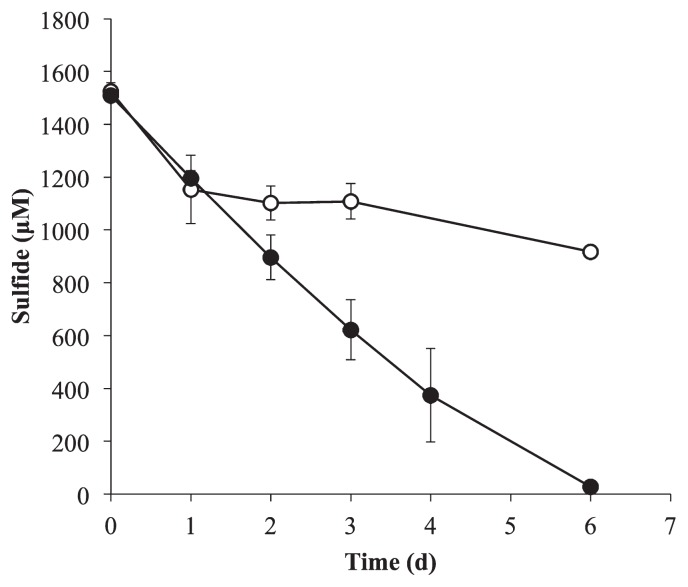
Sulfide consumption of *C. aggregans* strain ACA-12 during photoautotrophic growth with sulfide. Closed circle, sulfide concentrations with cells; open circle, sulfide concentrations without cells. Data are mean values of triplicate cultures±standard deviation.

Fig. 3 shows changes in sulfide concentrations under photoautotrophic conditions. Regardless of the presence or absence of cells, the concentration of sulfide in the culture decreased to 77% of the initial amount for 24 h due to abiotic auto-oxidation. The concentration of sulfide in the cell-free medium gradually decreased, but remained at >60% of the initial amount for 6 d. In cultures containing the cells, the amount of sulfide decreased to 2% of the initial amount for 6 d. Phase-contrast microscopy showed that strain ACA-12 produced small extracellular particles that were regarded as elemental sulfur (Fig. S1A). Elemental sulfur particles were also deposited through phototrophic sulfide oxidation by *C. aurantiacus* (19) and were similarly observed as bright particles under phase-contrast microscopy.

### Photoautotrophic growth with sulfide in *C. aggregans* strains MD-66^T^ and NBF

*C. aggregans* strains MD-66^T^ and NBF were previously isolated from Meotobuchi hot spring (11) and Nakabusa hot spring in Japan, respectively (21). In the present study, we also cultivated strains MD-66^T^ and NBF under photoautotrophic conditions with 1.0 mM sulfide. An increase in BChl *c* concentrations in the cultures of both strains was detected, and the growth rate was also calculated from exponential growth data plotted against time (data not shown). The cells of *C. aggregans* strain MD-66^T^ grew slightly under these conditions, and the growth rate was approximately 0.035 doublings h^−1^ (Table 2). Cell growth was not observed in media without sulfide. The small particles of elemental sulfur around grown cells were observed by phase-contrast microscopy (Fig. S1B). In the case of strain NBF, three transfers were performed in medium containing 1.0 mM sulfide, and in each case, BChl *c* concentrations were two- to six-fold higher than that in the medium without sulfide (*P*<0.01, *n*=7). The growth rate of strain NBF was approximately 0.043 doublings h^−1^.

## Discussion

Strain ACA-12 was isolated from Nakabusa hot spring via enrichment in an inorganic medium supplemented with sulfide. The phylogenetic analysis based on the 16S rRNA gene revealed that strain ACA-12 is closely related to *C. aggregans* and has the following properties that are similar to those of *C. aggregans*: a filamentous cell morphology and cell size, the presence of BChl *a* and *c*, and the formation of microbial mat-like cell aggregates in a liquid medium. Although our analysis based on 16S rRNA gene and ITS sequences did not distinguish the new strain from *C. aggregans* strain NBF (21) from the same hot spring, repetitive-sequence-based PCR DNA fingerprinting using BOX and ERIC primers clearly differentiated between these strains (Fig. 1). Based on physiological and genetic information, it is apparent that strain ACA-12 is a new strain of *C. aggregans*.

*C. aggregans* strain ACA-12 grew significantly under photoautotrophic conditions with sulfide and the cell yield estimated based on BChl *c* concentrations was in proportion to sulfide concentrations (Fig. 2). Sulfide concentrations decreased as cell growth progressed (Fig. 3), indicating that *C. aggregans* strain ACA-12 had the ability to use sulfide as an electron donor under photoautotrophic conditions. The growth rate of strain ACA-12 was 0.080 doublings h^−1^ (Table 2), which was four-fold faster than the previously recorded growth rate under photoautotrophic conditions for the well-studied strain, OK-70-fl, belonging to the closely related species, *C. aurantiacus* (19). Strain ACA-12 showing fast photoautotrophic growth was the first photoautotrophic strain in *C. aggregans* enriched from a natural hot spring using a sulfide-supplemented inorganic medium under photoautotrophic conditions.

Photoautotrophic growth ability was also investigated in the previously known *C. aggregans* strains MD-66^T^ (11) and NBF (21). These two strains showed slight sulfide-dependent photoautotrophic growth in the present study even though they are regarded as photoheterotrophs. However, their growth was clearly slow, with rates that were twice as slow as that of *C. aggregans* ACA-12 (Table 2). These results suggest that all of the *C. aggregans* strains tested possess photoautotrophy to some extent, and the present study is the first to demonstrate that photoautotrophic growth with sulfide is common in *C. aggregans*, previously regarded as a photoheterotroph.

Although we observed the photoautotrophic growth of *C. aggregans* strains MD-66^T^ and NBF, growth yields were lower than those under photoheterotrophic growth conditions. This may be one of the reasons why the photoautotrophic growth of strain MD-66^T^ was not detected in a previous study (11).

In Nakabusa hot spring, a microbial mat containing *C. aggregans* as a dominant phototroph is frequently found at approximately 65°C. The water at this hot spring contains a sufficient amount of sulfide (0.046–0.278 mM) to support the photoautotrophic growth of *C. aggregans* (17, 22). *C. aggregans* is widely distributed below the surface layer of a microbial mat (17). Since oxygen is consumed by aerobic bacteria in the surface layer, sub-surface layers are expected to become anaerobic, and, thus, photoautotrophic growth is possible. Strain NBF (which basically grows photoheterotrophically, but also shows slight photoautotrophy on sulfide) also thrived in the same hot spring with strain ACA-12. Although a detailed investigation of the distribution of closely related *C. aggregans* strains in natural environments has not yet been conducted, each strain appeared to have grown at sites under segregated conditions, *e.g*., with or without cyanobacteria and different temperatures.

Madigan and Brock (19) reported that deposited elemental sulfur was detected by phase-contrast microscopy in a culture of *C. aurantiacus* strain OK-70-fl. Similarly, we observed the deposition of bright particles in cultures of *C. aggregans* (Fig. S1). In the genus *Chloroflexus*, the presence of sulfur dioxygenase, which oxidizes elemental sulfur and converts it to sulfite, has not been reported, and *Chloroflexus* does not appear to have the ability to use elemental sulfur as an electron donor in a photosynthetic electron transport system.

The enzyme SQR is classified into six groups. The genomes of *Chloroflexus* species, including strain ACA-12, contain a gene for Type II SQR (Table 1). Type II SQR is widely present among cyanobacteria, non-phototrophs, and many eukaryotes (20). Although type II SQR is considered to have various functions, such as detoxification (27), it may be used for dissimilatory sulfide oxidation in the autotrophic growth of *Chloroflexus* species. The present study is the first to demonstrate the photoautotrophic growth on sulfide of strains of *C. aggregans* that contain the Type II SQR gene in their genomes. This may be indirect evidence for Type II SQR having the function of dissimilatory sulfide oxidation, similar to other types of SQR.

*C. aggregans* strains are considered to basically be photoheterotrophs that require organic compounds produced by neighboring cyanobacteria in natural environments. However, this may not be accurate because photoautotrophic growth was detected in *C. aggregans* strains in the present study. Therefore, *C. aggregans* is not just a consumer that depends on cyanobacteria, it may thrive independently with carbon fixing, and, hence, cannot be ignored as one of the primary producers in hot springs, as expected based on the biological activities of the microbial mats (17). However, photoautotrophic ability differs from strain to strain, as shown in this study. This ability may increase or decrease over time in natural hot springs with obligate photoautotrophic cyanobacteria.

## Supplementary material



## Figures and Tables

**Table 1:** Sequence differences between strain ACA-12 and other strains

	16S rRNA gene (1324 bp)	ITS region^a^ (182 bp)	Type II SQR (267 aa)
*C. aggregans* NBF	100%	100%	100%
*C. aggregans* MD66^T^	98.9%	91.8%	100%
*C. islandicus* isl-2^T^	97.2%	—	93.6%
*C. aurantiacus* J-10-fl^T^	95.4%	—	90.6%
*C. aurantiacus* OK-70-fl	95.1%	—	No sequence data
*C. aurantiacus* Y-400-fl	95.4%	—	90.6%
*Chloroflexus* sp. Y-396-1	95.3%	—	95.5%
*Chloroflexus* sp. MS-G	95.2%	—	95.5%

The accessions of the 16S rRNA gene, ITS region, and Type II SQR of strains of *C. aggregans* are as follows: *C. aggregans* ACA-12, LC438939, LC439412, and LC439413; *C. aggregans* NBF, LC439414, LC439415, and LC439416; *C. aggregans* DSM9485^T^, NR_074226.1, CP001337.1: c4138337-4138519, and ACL22998.1. The accessions of the 16S rRNA gene and Type II SQR of other *Chloroflexus* strains are as follows: *C. islandicus* isl-2^T^, KP939041.2 and OAN46183.1; *C. aurantiacus* J-10-fl^T^, CP000909.1:c780400-778922 and YP_001637460.1; *C. aurantiacus* Y-400-fl, NC_012032.1:c780547-779061 and ACM55565.1; *Chloroflexus* sp. Y-396-1, NZ_KI911784.1:c2937687-2936196 and WP_028460186.1; *Chloroflexus* sp. MS-G, JPIM01000237.1 and WP_031458311.1.

The accession number of the 16S rRNA gene of *C. aurantiacus* OK-70-fl is AJ308500.1.

a The identity of ITS was calculated among *C. aggregans* strains.

**Table 2:** Capability of photoautotrophic growth with sulfide in *Chloroflexus* strains

	Doublings h^−1^^a^	Genome available	Sampling site
*C. aggregans* ACA-12	0.080	−	Nakabusa hot spring, Japan (this study)
*C. aggregans* NBF	0.043	−	Nakabusa hot spring, Japan (21)
*C. aggregans* MD-66^T^	0.035	+	Meotobuchi hot spring, Japan (11)
*C. aurantiacus* OK-70-fl	0.022	−	Kah-Nee-ta hot springs, Oregon, USA (24)
*Chloroflexus* sp. MS-G	—b	+	Mushroom Spring, Yellow stone national park, USA (28)

a The doublings h^−1^ of strains of *C. aggregans* was estimated in this study. Strain ACA-12 grew photoautotrophically with 1.5 mM sulfide, and strain NBF and MD-66T grew photoautotrophically with 1.0 mM sulfide. The doublings h^−1^ of *C. aurantiacus* OK-70-fl was calculated using the graph of autotrophic growth of this strain in a study by Madigan and Brock (19). Cells were grown anaerobically in inorganic media containing minerals, vitamins, nitrite, and ammonium salt as nitrogen sources, bicarbonate as a sole carbon source, and 2.1 mM sulfide (50°C and light of 548 lx). We defined the amount of BChl from the graph for calculations; days 1 and 6 were 0.8 and 4.8 μg mL^−1^, respectively.

b Autotrophic growth was observed in the agar deep culture containing sulfide and inorganic carbon.
