# Supplementary material for: Sulfide-dependent Photoautotrophy in the Filamentous Anoxygenic Phototrophic Bacterium, Chloroflexus aggregans
Source: Microbes Environ. 2019 Aug 8;34(3):304–9. doi: 10.1264/jsme2.ME19008 (PMC6759344; doi:10.1264/jsme2.ME19008)
Supplement: Supplementary file 1 [file 34_304_s1.pdf]

Supplementary material

Sulfide-dependent photoautotrophy in a filamentous anoxygenic phototrophic bacterium, *Chloroflexus aggregans*

Nanako Kanno, Shin Haruta and Satoshi Hanada

Department of Biological Sciences, Graduate School of Science, Tokyo Metropolitan University, 1-1 Minami-Osawa, Hachioji, Tokyo 192-0397, Japan

A

B

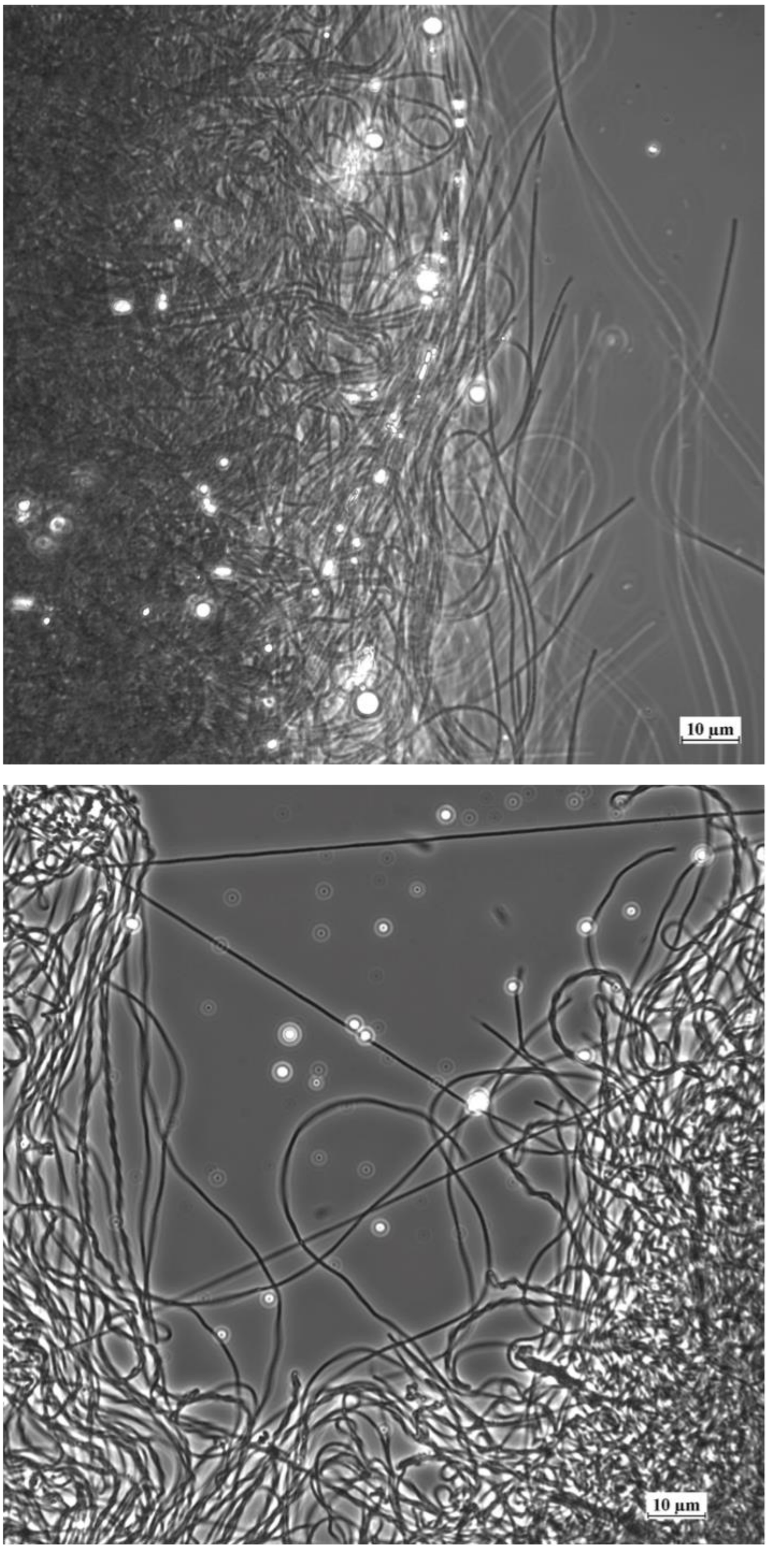

Fig. S1. Phase contrast photomicrograph of the culture during photoautotrophic growth with 1.5 mM sulfide in *C. aggregans* strain ACA-12 (6 days culture) (A) and strain MD-66^{T} (4 days culture) (B). Marker bar represents 10 μm.

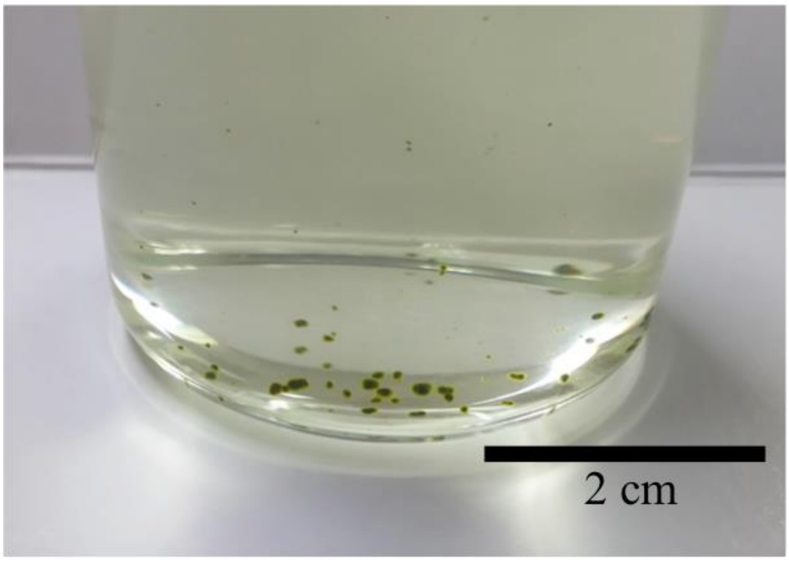

Fig. S2. Sticky cell aggregates of *C. aggregans* strain ACA-12 under photoautotrophic growth with sulfide.

Cells were grown in 100 mL vial containing 40 mL media with 1.5 mM sulfide.

Photograph shows cell aggregates of 5-d old culture solution in the glass vial.

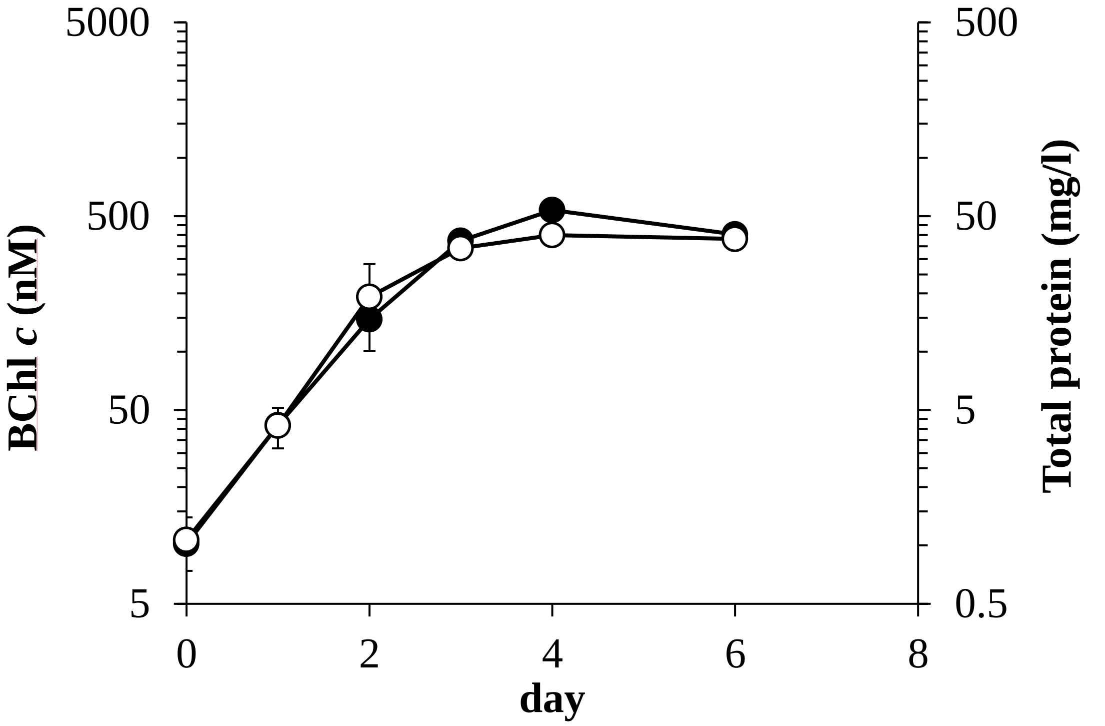

Fig. S3 Change over time of BChl *c* and total protein in culture under photoautotrophic conditions with 1.5 mM sulfide.

Closed circle, BChl *c*; open circle, total protein.

In each time, all cells in vial were harvested and pigment and protein were extracted form the same vial. Data are mean values of triplicate trials ± standard deviation.

Culture conditions

Cells were cultured in 100 mL-vial containing 40 mL of inorganic medium anaerobically at 55°C under incandescent illumination (approximately 230 W/m^2).

The inorganic medium (pH 7.0) was prepared as follow: the vial containing the solution consisted of (per liter) 0.1 g $(NH_4)_2SO_4$, 0.15 g KH_2PO_4, 0.16 g K_2HPO_4, 0.2 mL of a vitamin mixture (1), and 2 mL of a basal salt solution (1) was gassed with a mixture of N_2 and CO_2 (80%:20% [v/v]) and then autoclaved. The gas phase of the vials was replaced with same gas composition mixture. Prior to inoculation, filter-sterilized $NaHCO_3$ solution (final concentration, 50 mM) and Na_2S solution (final concentration, 1.5 mM) were added to the vial.

Extraction and quantification of BChl *c* and total protein:

Cells in the vial were harvested onto a filter (0.2 μm pore size, hydrophilic polycarbonate membrane, 25 mm diameter) and resuspended them in 1 mL of PBS with 0.1% Tween 80. The cells were dispersed using by sonication in a sonication bath for 10 min at 4°C (15 sec sonication and 15 sec interval, 20 KHz/160 W, Bioruptor UCD-200T, Cosmobio Corp., Tokyo, Japan).

BChl *c* content was determined by extraction with acetone-methanol (cell suspension:aceton:methanol = 1:7:2). Mixture was centrifuged (13,982 × g, 5 min, 4°C) and supernatant was collected. Absorption spectrum was recorded between 400 and 1,000 nm using a UV-1800 spectrophotometer (Shimadzu, Kyoto, Japan). For calculation of BChl *c* (absorbing at 666 nm) concentration, an extinction coefficient of 74 mM^{-1} cm^{-1} (2) was used.

For measurement of total protein, the cell suspension was heated at 95°C for 15 min with 2% SDS. Suspension was centrifuged (13,982 × g, 5 min, room temperature) and supernatant was collected. Protein content in supernatant was determined by using the DC protein assay (bovine serum albumin was used for standard) (Bio-Rad Laboratories, Inc., Hercules, CA, USA).

Vials were triplicated for this experiment.

References

1, Hanada, S., A. Hiraishi, K. Shimada, and K. Matsuura. 1995. *Chloroflexus aggregans* sp. nov., a filamentous phototrophic bacterium which forms dense cell aggregates by active gliding movement. Int. J. Syst. Bacteriol. 45:676-681.

2, Feick, R.G., M. Fitzpatrick, and R.C. Fuller. 1982. Isolation and characterization of cytoplasmic membranes and chlorosomes from the green bacterium chloroflexus aurantiacus. J. Bacteriol. 150:905-915.
